# Echo reception in group flight by Japanese horseshoe bats, *Rhinolophus ferrumequinum nippon*

**DOI:** 10.1098/rsos.211597

**Published:** 2022-02-09

**Authors:** Kazuma Hase, Yukimi Kadoya, Yuki Takeuchi, Kohta I. Kobayasi, Shizuko Hiryu

**Affiliations:** ^1^ Graduate School of Environmental Studies, Nagoya University, Furo-cho, Chikusa-ku, Nagoya 464-8601, Japan; ^2^ Faculty of Life and Medical Sciences, Doshisha University, 1-3 Tatara miyakodani, Kyotanabe, Kyoto 610-0321, Japan; ^3^ Research Fellow of Japan Society for the Promotion of Science, 5-3-1 Kojimachi, Chiyoda-ku, Tokyo 102-0083, Japan

**Keywords:** auditory fovea, echolocation, Doppler shift compensation, jamming avoidance

## Abstract

The ability to detect behaviourally relevant sensory information is crucial for survival. Especially when active-sensing animals behave in proximity, mutual interferences may occur. The aim of this study was to examine how active-sensing animals deal with mutual interferences. Echolocation pulses and returning echoes were compared in spaces of various sizes (wide and narrow) in *Rhinolophus ferrumequinum nippon* flying alone or in a group of three bats. We found that in the narrow space, the group-flying bats increased the duration and bandwidth of the terminal frequency-modulated component of their vocalizations. By contrast, the frequency of the returning echoes did not differ in the presence of conspecifics. We found that their own echo frequencies were compensated within the narrow frequency ranges by Doppler shift compensation. By contrast, the estimated frequencies of the received pulses emitted by the other bats were much more broadly distributed than their echoes. Our results suggest that the bat auditory systems are sharply tuned to a narrow frequency to filter spectral interference from other bats.

## 1. Introduction

Living organisms need to process biologically relevant information and then adapt their behaviour for survival. As the world is full of information, biologically relevant information is often embedded in other types of information, called noise. Animal perception using active-sensing can be negatively affected by noise from nearby conspecifics when animals behave in proximity. Under such circumstances, they must filter their own signals from those emitted by conspecific animals, which have similar spectral and temporal features. Echolocating bats process echoes of self-generated ultrasounds to perceive surroundings in the dark. They are supposed to be exposed to many conspecific sounds because they often behave with multiple conspecifics [1–6]. It remains unknown how echolocating bats extract their own echoes in the presence of acoustic interferences by conspecific sounds, so-called jamming [3].

Several studies have demonstrated that frequency-modulating (FM) bats show spectral jamming avoidance response (JAR), where they shift the frequency of their echolocation pulses, to avoid spectral overlap during group flight [7–12] or when they are exposed to noises [13–18]. Telemetry sound recordings revealed that *Miniopterus fuliginosus* flying in a group of four bats shifted the terminal frequencies of their emitted pulses away from each other [9]. However, whether spectral JAR is useful for mitigating jamming is still unknown. Bats fly fast enough to induce the Doppler shifts in pulses and echoes, so we assume that the Doppler effect influences how bats interfered with each other, especially those with auditory systems extremely sensitive to Doppler shifts.

Doppler shift compensation (DSC) is a highly sophisticated audio-vocal behaviour displayed by some species of bats from the Rhinolophidae [19–22], Hipposideridae [22–25] and Mormoopidae families [26–28]. These bats emit a combination of a relatively long constant-frequency (CF) component preceded and followed by a short FM component. They adaptively change the dominant second harmonic CF component (CF2) of the emitted pulse to cancel the flight-induced Doppler shift in their echo CF2 [29]. The compensated echo CF2 is called the reference frequency. The cochlea of these bats is extremely sensitive to echoes decreasing to a narrow frequency range, called auditory fovea [30].

Doppler shift-compensating bats seem not to employ spectral JAR under acoustically jammed conditions. In the presence of conspecific or heterospecific bats, *Rhinolophus capennsis* did not change the pulse CF2 frequency but changed the duration of their FM component and the bandwidth of their CF-FM pulses [31]. Similarly, Jones *et al*. [32] found no systematic changes in the pulse CF2 frequency in *Hipposideros speoris* in the presence of conspecifics [32]. To our best knowledge, however, only Furusawa *et al*. [33] measured echoes received by bats during group flight [33]. The authors demonstrated that during paired flights, *R. ferrumequinum nippon* did not increase but even decreased individual differences in the reference frequency. They proposed that individual differences in the reference frequency ranging from 0.0 to 1.2 kHz are still larger than the frequency discriminability of approximately 70 Hz, which was estimated from the data of other CF-FM bat species [34,35]. The apparent lack of spectral JAR in the horseshoe bats can be due to the auditory fovea with fine resolution at the reference frequencies. However, knowledge on how pulses of other bats interfere with another bat's echoes when the bat flies with multiple conspecifics is still limited. This is because the frequency of other bat sounds that a bat hears can be changed by the relative velocity among individual bats. Therefore, echo measurement is required for closer examination of how the auditory system of bats processes weak echoes in the presence of noisy conspecifics.

In this study, we hypothesized that Doppler shift-compensating bats are tolerant to conspecific jamming in terms of the reference frequency for the following reasons: (i) in addition to the individual difference in the reference frequency, the frequency of the pulses of other bats that is received by another bat during group flight can dynamically change owing to the Doppler effect and amount of DSC; (ii) the bats have highly specialized auditory systems with an extreme spectral resolution at the individual-specific reference frequency. However, whether these dynamic changes enable auditory fovea to filter out sounds of other bats remains equivocal because studies have not assessed the relationship between bats' own echoes and the pulses of other bats that individual bats receive.

The purpose of the present study was to examine how *R. f. nippon* change the acoustic characteristics of emitted pulses and returning echoes to mitigate jamming from each other. For this purpose, by attaching telemetry microphones to individual bats in a group of three bats, we recorded echolocation pulses and their echoes. We compared the acoustic characteristics of the pulses and echoes between single and group flights. We also changed the sizes of the flight spaces to investigate how the density of conspecifics flying together influenced their behaviours. Moreover, to investigate whether Doppler shift-compensating bats are tolerant to conspecific jamming in terms of the reference frequency, we also investigated the relationships of the frequency of bats' own echoes and the pulses from other bats in the group as estimated from the relative velocity between individual bats.

## 2. Methods

### 2.1. Subjects

Nine adult Japanese horseshoe bats (*R. f. nippon*, six males and three females) were caught from wild colonies in artificial caves in Fukui Prefecture, Japan.

The echolocation sounds emitted by the bats consist of a relatively long CF portion and are accompanied by a brief upward initial FM (iFM) component and a brief downward terminal FM (tFM) component. The bats emit multi-harmonic echolocation sounds, of which the second harmonic of the CF components (CF2) is the most prominent. The iFM component is often very weak or sometimes even absent. The bats exhibit a DSC behaviour to compensate for the echo CF2 frequency, which slightly varies between individuals in a colony [33].

### 2.2. Experimental procedure

All the experiments were conducted in an experimental chamber (9 × 4.5 × 2.4 m) at Doshisha University in Kyoto, Japan. The chamber was constructed with steel plates to reduce interference from external electromagnetic noise and commercial FM radio stations. To assess how the reference frequency changed during group flight, acoustic foam was not used to ensure that the chamber was echoic. This allowed us to record echoes from the walls, which made it easier to estimate the reference frequencies.

*Epitescus fuscus* flying in a pair broaden individual differences in acoustic characteristics of emitted pulses as the inter-bat distance decreases [36]. Therefore, we created flight spaces that varied in size, so that distances between bats in a group could be constrained, which allowed us to examine how their echolocation behaviour during group flight changed in the different levels of acoustic interferences. The wide space (6 × 4.5 × 2.4 m) was constructed with a single net suspended from the ceiling, 6 m from the front wall (electronic supplementary material, figure S1A). The narrow space (2 × 4.5 × 2.4 m) was constructed by adding a suspended net to the wide chamber (electronic supplementary material, figure S1B). A telemetry microphone [20,37] was attached to the back of each bat to separately record emitted pulses and returning echoes belonging to all individuals flying together in a group. During all the experiments, a long-wavelength light with filters (removing wavelengths less than 650 nm) was used to minimize visual effects on the bats [38].

We randomly assigned nine bats to each of the 12 groups consisting of three bats each, with some overlaps. Of the 12 groups, six were tested in the wide space, and the other six were tested in the narrow space. Because the bats tended to stop flying and land on the walls or the nets typically in 30 s, for each flight space, the bats were tested under three experimental conditions as follows: single flight 1, group flight and single flight 2. First, an experimenter released each bat in each group, so that the bat flew in the experimental space for approximately 30 s (single flight 1). After the single flights were recorded, we kept the bats individually in cages. Then, two experimenters released three bats, so that they flew together in the space for approximately 60 s (group flight). Finally, again, an experimenter allowed each bat in each group to fly alone in the space for approximately 30 s (single flight 2). This procedure did not enable us to monitor dynamics of changes in acoustic characteristics of pulses and echoes emitted by bats in response to other bats. It, however, allowed the bats to accomplish each flight condition much more easily. For each group, all the flights were conducted within 1 d to maintain a stable reference frequency, because the reference frequency can change on a daily basis [33].

### 2.3. Video recordings

The three-dimensional flight trajectory of the bats was calculated by recording flight using two digital video cameras (30 fps; MotionXtra NX8-S1, IDT Japan, Inc., Tokyo, Japan) with Motion Studio v. 2.12.6.0 (IDT Japan, Inc.). The cameras were located outside the flight space at the top corners of the chambers. The video images were analysed using a motion capture software (DippMotion PRO v. 2.21a, Ditect Corporation, Tokyo, Japan). The three-dimensional position coordinates of each bat were calculated using the direct linear transformation method, deriving the bat's position from the parallax view of the camera images obtained from two directions. The images captured with the video cameras, and the sounds measured using telemetry microphones were synchronized with the trigger signal manually generated by an experimenter.

### 2.4. Sound recordings

The echolocation pulses and echoes of each bat were separately recorded using a custom-made miniature telemetry microphone mounted on the bat's back [20,37]. The telemetry microphone consisted of a 1/8 inch omnidirectional condenser microphone (Knowles, Model FG-23329-C05, Itasca, IL, USA), a custom-made transmitter circuit, a 1.5 V hearing aid battery (Sony, Type SR626SW, Tokyo, Japan) and a transmitting antenna. The telemetry microphone weighed approximately 0.6 g including the battery and could last for approximately 10 min with a single battery. To attach the telemetry microphone onto the back of a bat, we used a piece of double-sided adhesive tape. The adhesive tape was removed using a parting agent after the experiments.

The detailed procedure for recording the sounds of multiple bats has been described previously [9]. The telemetry microphone transmits FM radio signals using a carrier frequency between 76 and 104 MHz. A different carrier frequency was assigned to each telemetry microphone in each group to avoid interference between the transmitted signals. After an FM radio antenna (Terk Technologies Corporation, FM+, Commack, New York, USA) suspended from the ceiling of the chamber received the signals, these were demodulated using a custom-made FM receiver (ArumoTech Corporation, Kyoto, Japan) with bandpass filters of 10–200 kHz. The signals were then digitized using a high-speed data acquisition card (16-bit, *f*_s_ = 500 kHz; Model NI PXI-6358, National Instruments, Austin, TX, USA).

### 2.5. Sound analysis

The echolocation pulses and echoes were manually analysed from the telemetry microphone recordings on spectrograms by using custom-written Matlab scripts. Pulse duration, bandwidths and tFM durations were measured on the spectrogram. The CF2 frequencies of the pulses and echoes were calculated from a visually selected area around the CF component by a fast Fourier transformation of 16 384 sample points. The analysis revealed a frequency resolution of approximately 31 Hz. In the present study, we defined the reference frequency of each bat as the mean CF2 frequency of echoes that were recorded with a telemetry microphone. As bats typically fly in a circular path in flight spaces, the walls from which echoes were returning were difficult to identify. This probably led to the difficulty in extracting the compensated echoes in the experiments in the wide spaces (please refer to §3). We defined the silent time as the interval between sonar sound groups, which are grouped pulses emitted in short interpulse intervals.

During group flight, telemetry microphones sometimes recorded the pulses of other individual bats besides those of the focal bat. To extract the echolocation pulses of the focal bat, visual inspection was used on the basis of the differences in power and timing across the spectrograms of the three recorded channels that occurred owing to the spatial relationships of the individual bats.

### 2.6. Estimating the frequencies of the pulses emitted by the other bats during group flight

In the experiments, the telemetry microphone carried by each bat during group flight scarcely recorded the pulses emitted from the other bats. Therefore, to determine the relationships of the frequencies between the echoes and the other bats' pulses that the focal bat may hear, we estimated the frequency of the other bats' pulses from the relative velocity between individuals, which were calculated from flight trajectories. The flight trajectories were fitted with 10th order polynomials to estimate the position of pulse emissions by bats using Igor Pro (v. 5.0.3; WaveMetrics Inc., Lake Oswego, OR, USA). Each coordinate was divided into two parts (5 s and 5 s sequences), which were then fitted with the polynomials. First, we calculated the flight directions of the two focal bats.

2.1 $$\;\begin{array}{c} \theta_{An}=\tan^{-1}\frac{y_{An}-y_{An-1}}{x_{An}-x_{An-1}}, \end{array}$$

2.2 $$\;\begin{array}{c} \theta_{Bn}=\tan^{-1}\frac{y_{Bn}-y_{Bn-1}}{x_{Bn}-x_{Bn-1}} \end{array},$$

2.3 $$\;\begin{array}{c} \varphi_{An}=\tan^{-1}\frac{z_{An}-z_{An-1}}{x_{An}-x_{An-1}} \end{array}$$

2.4 $$\mathrm{and}\;\begin{array}{c} \;\varphi_{Bn}=\tan^{-1}\frac{z_{Bn}-z_{Bn-1}}{x_{Bn}-x_{Bn-1}}, \end{array}\;$$

where $\theta_{A}$ and $\theta_{B}$ are the flight directions in the *x*–*y* plane of bats A and B, respectively; $\varphi_{A}$ and $\varphi_{B}$ are the flight directions in the *x*–*z* plane of bats A and B, respectively; and (*x*_A_, *y*_A_, *z*_A_) and (*x*_B_, *y*_B_, *z*_B_) are the three-dimensional coordinates of bats A and B, respectively. The subscript *n* indicates the number of frames. Then, we calculated the angle between the bats as follows:

2.5 $$\begin{array}{c} \theta_{\mathrm{AB}n}=\tan^{-1}\frac{y_{Bn}-y_{An}}{x_{Bn}-x_{An}} \end{array}$$

and

2.6 $$\begin{array}{c} \varphi_{\mathrm{AB}n}=\tan^{-1}\frac{z_{Bn}-z_{An}}{x_{Bn}-x_{An}}, \end{array}$$

where $\theta_{\mathrm{AB}}$ and $\varphi_{\mathrm{AB}}$ are the angle between the bats in the *x*–*y* and *x*–*z* planes, respectively. We calculated the velocity of bat B relative to that of bat A.

2.7 $$\begin{array}{rl} v_{BAn} & =v_{Bn}\times \cos(\theta_{Bn}-\theta_{\mathrm{AB}n})\times \cos(\varphi_{Bn}-\varphi_{\mathrm{AB}n})-v_{An}\times \cos(\theta_{An}-\theta_{\mathrm{AB}n})\times \cos(\varphi_{An}-\varphi_{\mathrm{AB}n}), \end{array}$$

where $v_{BA}(t)$ is the velocity of bat B relative to that of bat A. Finally, we calculated the CF2 frequency of the pulse of bat B that was audible to bat A as follows:

2.8 $$\begin{array}{c} CF2_{\mathrm{BA}n}=CF2_{Bn}\times \frac{C}{C-\;|v_{BAn}|}, \end{array}$$

where $CF2_{\mathrm{BA}}$ is the CF2 frequency of the pulse of bat B that was audible to bat A, $\;CF2_{B}$ is the CF2 frequency of the pulse emitted by bat B, and *C* is the sound velocity in the air.

### 2.7. Statistical analysis

To examine whether acoustic characteristics of echolocation pulses and echoes were affected by other individuals and sizes of the flight spaces, we used linear mixed models (LMMs). The response variables were tFM bandwidth, tFM duration, pulse duration, silent time and s.d. of the reference frequency. The fixed effects were the flight condition (i.e. single flight 1, group flight, or single flight 2), the flight space (i.e. narrow or wide) and interaction of these two effects except for the s.d. of the reference frequency model. Because distributions of s.d. of the reference frequency were significantly different across the flight spaces, we built a separate model for each flight space. In the s.d. of the reference frequency model, the fixed effect was the flight condition. In all the models, bat ID was included as a random effect.

We qualified the model fit by plotting residuals. We used a type II Wald *χ*^2^-test to determine the significance of flight condition, flight space and interaction between flight condition and flight space within the models. If the main effects of flight condition were significant, *post hoc* tests were performed using the Tukey–Kramer method to correct for multiple comparisons.

All statistical analyses were performed using the statistical platform R (v. 4.0.3) [39]. We used the lmer function of the lme4 package (v. 1.1.26) [40] for the LMMs and the analysis of variance function of the car R package (v. 3.0.10) [41] for the type II Wald-χ^2^-test. We used the simulateResiduals and testDispersion functions of the DHARMa package (v. 0.4.2) [42] to check uniformity, dispersion and residuals of each model. The emmeans function of the emmeans package (v. 1.4.8) [43] was used for the *post hoc* analysis. *p*-values less than 0.05 were considered significant. Results are presented as mean ± s.d., unless otherwise stated.

## 3. Results

### 3.1. Changes in the acoustic characteristics of the echolocation pulses

We successfully recorded echolocation sounds and flight trajectories from three Japanese horseshoe bats flying together in the experimental chamber. The inter-individual distances and flight speeds of the bats in group flight were different between the narrow and wide spaces, indicating that different acoustically jammed conditions could be constructed (electronic supplementary material, figure S1C,D). The mean distance between the bats during group flight was shorter in the narrow space (1.8 ± 0.9 m) than in the wide space (2.7 ± 1.2 m; electronic supplementary material, figure S1C). The mean flight velocity was lower in the narrow space (2.2 ± 0.8 m s^−1^) than in the wide space (2.7 ± 0.7 m s^−1^; electronic supplementary material, figure S1D).

**Figure 1.**
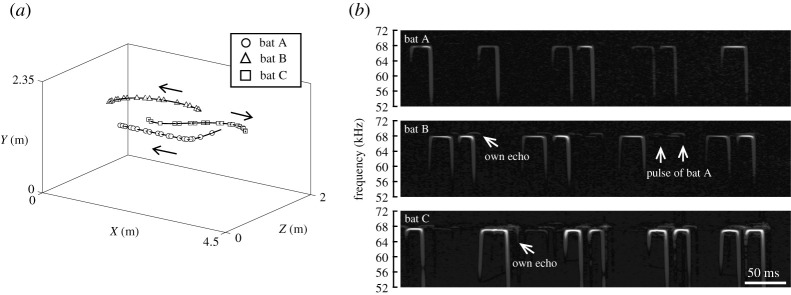
Group flight of three Japanese horseshoe bats, *R. f. nippon*. (*a*) Representative flight trajectories. Plots on the trajectories represent the locations of the echolocation sounds emitted by the bats. (*b*) Spectrogram of the sounds emitted by individual bats recorded with a telemetry microphone. The telemetry microphone recorded not only the bats' pulses and returning echoes but also other bats' sounds.

**Figure 2.**
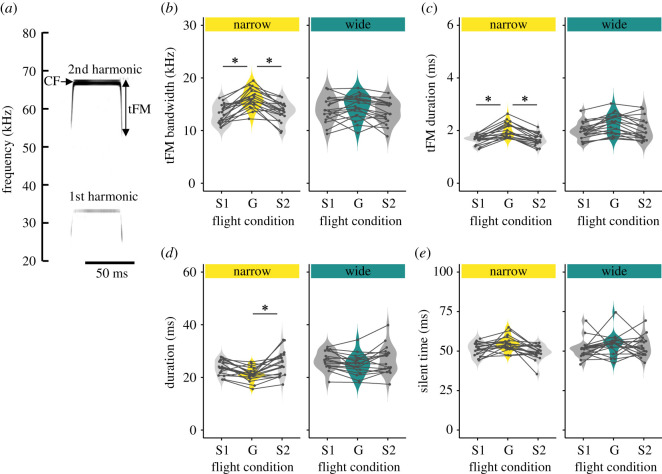
(*a*) Representative pulses emitted by *R. f. nippon*. Changes in tFM bandwidth (*b*), tFM duration (*c*), pulse duration (*d*) and silent time across the flight conditions (single flight 1, group flight and single flight 2) in the wide and narrow spaces. The bats increased their tFM bandwidth and tFM duration and decreased their pulse duration during group flight in the narrow space (Tukey–Kramer post hoc analysis, *p* < 0.05). No significant changes in pulse features were observed across the flight conditions in the wide space (Tukey–Kramer *post hoc* analysis, *p* > 0.0891). Violin plot illustrates kernel density of each acoustic characteristic for each flight condition. Black plots show mean values of acoustic characteristics of each individual.

Figure 1 shows the representative flight trajectories and spectrograms of emitted pulses and returning echoes recorded with the telemetry microphone carried by each bat during the group flight of three bats. The microphones recorded the pulses and echoes of other bats in addition to those of the focal bat, which indicated sound transfer between individuals during group flight. The bandwidth of the tFM component (tFM bandwidth; figure 2*a*) was not significantly different among single flight 1 (14.1 ± 2.4 kHz), single flight 2 (13.5 ± 2.1 kHz) and group flight (14.5 ± 2.3 kHz) in the wide space (Tukey–Kramer *post hoc* analysis, *p* > 0.2798; figure 2*b*). On the other hand, the tFM bandwidth was significantly changed from single flight 1 (13.1 ± 1.8 kHz) and single flight 2 (13.5 ± 1.9 kHz) to the group flight (16.1 ± 1.9 kHz) in the narrow space (Tukey–Kramer *post hoc* analysis, *p* < 0.05; figure 2*b*). tFM bandwidth was not significantly changed from single flight 1 and single flight 2 space (Tukey–Kramer *post hoc* analysis, *p* = 0.9640; figure 2*b*). Moreover, the duration of the tFM component (tFM duration) was not significantly different among single flight 1 (2.0 ± 0.3 ms), single flight 2 (2.0 ± 0.4 ms) and group flight (2.3 ± 0.4 ms) in the wide space (Tukey–Kramer *post hoc* analysis, *p* > 0.1152; figure 2*c*). The tFM duration was significantly changed from single flight 1 (1.7 ± 0.2 ms) and single flight 2 (1.7 ± 0.2 ms) to the group flight (2.0 ± 0.3 ms) in the narrow space (Tukey–Kramer *post hoc* analysis, *p* < 0.05; figure 2*c*), but not significantly different between single filght 1 and single flight 2 (Tukey–Kramer *post hoc* analysis, *p* = 1.0000; figure 2*c*).

The duration of the CF-FM pulses (pulse duration) was not significantly different among single flight 1 (26.5 ± 3.5 ms), single flight 2 (24.7 ± 4.0 ms) and group flight (26.8 ± 5.3 ms) in the wide space (Tukey–Kramer *post hoc* analysis, *p* > 0.3612). The pulse duration was not significantly changed from single flight 1 (23.8 ± 2.8 ms) to the group flight (21.1 ± 2.6 ms) and single flight 2 (25.3 ± 4.7 ms) (Tukey–Kramer *post hoc* analysis, *p* > 0.1171; figure 2*d*) but was significantly different between the group flight and single flight 2 in the narrow space (Tukey–Kramer *post hoc* analysis, *p* < 0.05; figure 2*d*).

In addition to the changes in the spectrotemporal characteristics of the echolocation sounds, we also examined changes in the temporal patterns of the emissions. Figure 2*e* shows the changes in silent time across all flight conditions. The silent time was not significantly different among single flight 1 (51.3 ± 6.1 ms), single flight 2 (52.8 ± 6.4 ms) and group flight (54.1 ± 7.1 ms) in the wide chamber (Tukey–Kramer *post hoc* analysis, *p* > 0.6249; figure 1*e*). Also, silent time was not significantly changed from single flight 1 (51.0 ± 4.3 ms) and single flight 2 (49.2 ± 4.6 ms) to the group flight (54.8 ± 4.8 ms) in the narrow space (Tukey–Kramer *post hoc* analysis, *p* > 0.0891; figure 2*e*).

### 3.2. Changes in the reference frequency of the returning echoes

**Figure 3.**
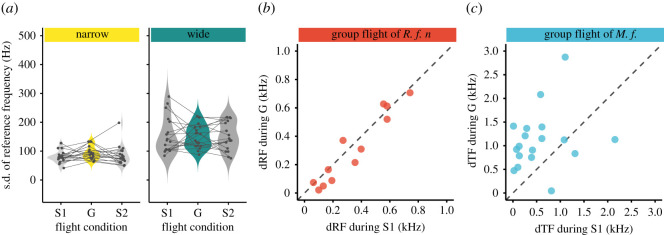
(*a*) Changes in the s.d.s of the reference frequencies across the flight conditions in the wide and narrow spaces. The s.d.s did not significantly change across the flight conditions (Tukey–Kramer *post hoc* analysis, *p* > 0.4437). (*b*) Relationships of the ΔRFs of the bats between the single and group flights in the narrow space. (*c*) Relationships of the delta terminal frequencies (ΔTFs) emitted by *Miniopterus fuliginosus* bats between the single and group flights in the narrow space, adapted from a previous study [9].

We examined how the reference frequency was changed in group flight when the bats performed DSC (figure 3*a*). We found no significant difference in the s.d.s of the reference frequency across the single and group flights in either the wide or narrow space (type II Wald *χ*^2^-test, *χ*^2^ = 351.22, d.f. = 1, *p* > 0.4437; figure 3*a*), which suggests that the bats performed DSC in the group flight as accurately as in the single flights. In the wide space, the s.d. of the reference frequency was 154 ± 63 Hz in single flight 1, 150 ± 40 Hz in group flight and 152 ± 50 Hz in single flight 2. In the narrow space, the s.d.s were 80 ± 21, 93 ± 23 and 85 ± 34 Hz, respectively. The mean s.d. of the reference frequency in the wide space was almost twice as large as that in the narrow space, consistent with the values from previous studies on the same species (approximately 60–70 Hz) [20,33]. Larger s.d.s could not reflect reference frequencies because of the echoes reflected from multiple walls owing to the circular flight path in the wide space. Therefore, data from the wide space were excluded in further analysis.

Next, we investigated how reference frequencies differed between the flight conditions. We defined ΔRF as the difference in mean reference frequency between the two bats that were closest in terms of their reference frequencies. No clear pattern in ΔRF was observed (figure 3*b*). The frequency-shifting jamming avoidance observed in the FM bat species, *Miniopterus fuliginosus*, in our previous study [9] was not observed in the present study (figure 3*b,c*). This suggests that CF-FM bats use a different mechanism to mitigate jamming.

### 3.3. How bats could be potentially jammed by other bats in terms of the reference frequency

**Figure 4.**
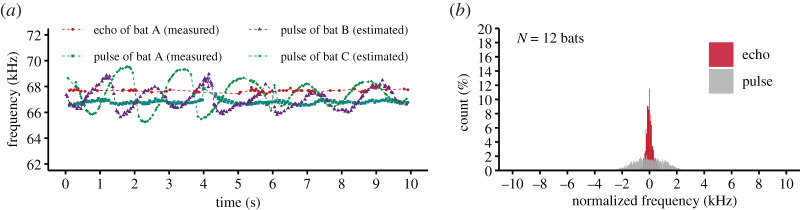
Relationships between a bat's reference frequency and the frequencies of the pulses it received from other bats. (*a*) Changes in a bat's echo CF2 frequency (measured) and the pulse frequencies it received from the other bats (estimated) during group flight. (*b*) Distribution of the frequencies of a bat's own echoes and the pulses it received from the other bats during group flight. The values were normalized by the bat's own reference frequency.

To understand how bats were jammed by other bats in terms of the reference frequency, we estimated the frequency of the other bats' pulses from the relative velocity between individuals, which were calculated from flight trajectories. This estimation was applied for 12 bats in four groups in the narrow space because we could not obtain the video data from the rest of the six groups tested in the narrow space. Figure 4*a* shows the representative changes in pulse frequency and returning echoes of one bat in group flight and the frequencies of pulses the bat received from the other two bats in the present study. The frequencies of the pulses the bat received from the other bats dramatically changed across time as compared with the echo frequencies of the bat. Figure 4*b* shows a histogram of the normalized frequencies received by a bat. The frequencies were normalized by subtracting its reference frequency from those of its echoes and estimates of the other group members' frequencies that were perceived by the bat. The estimated frequencies of the other bats' sounds were much more broadly distributed than the echoes actually received by the bat.

## 4. Discussion

In the present study, we recorded echolocation pulses and their returning echoes generated by *R. f. nippon* during group flight. We found that the bats decreased their pulse duration and increased the duration and bandwidth of the tFM component of the emitted pulses while flying in groups in the narrow space. These changes might be adaptations to the acoustic interferences by conspecifics. Nevertheless, the bats performed the DSC as accurately as in the single flight and did not change their reference frequency in the presence of conspecifics, which suggests that CF-FM bats have no clear frequency-shifting JARs.

Another CF-FM bat species, *R. capensis*, increased the duration and bandwidth of their tFM component during paired flight [31]. Similarly, stationary *R. ferrumequinum* broadened the bandwidth of the FM component of their CF-FM pulses in response to band-limited noise [44]. A previous study suggested that *R. ferrumequinum* calculate time differences in tFM components between pulses and echoes to measure distances by echolocation [45]. Besides, prolonging the duration is thought to improve the signal-to-noise ratio of echoes to the corresponding pulse emissions [46,47]. These findings suggest that in the presence of conspecifics, bats improve their ranging performance by highlighting their tFM components to avoid collision and/or capture insects successfully.

In the present study, pulse duration of the emitted pulses was shorter in group flight than in single flights. Similarly, *R. capensis* decreased their pulse duration during paired flight [31]. For the DSC in horseshoe bats, temporal overlaps between pulses and echoes are necessary [48]. Maximum temporal overlap between pulses and echoes is determined by pulse duration. Therefore, pulse duration could reflect how far they perceive by echolocation. This implies that bats would shorten time windows for echo reception by decreasing pulse duration to avoid confusing by other bats' sounds.

Our results suggest that the frequencies of other bats' sounds can change dramatically owing to the Doppler effect and DSC themselves (figure 4). Individual differences in the reference frequency in a colony are typically as small as 0.1 kHz [33]. CF-FM bats have a highly specialized auditory system that is sharply tuned to their reference frequency, called the auditory fovea [30]. The auditory systems of *R. f. nippon* bats are tuned within fairly narrow ranges corresponding to individual-specific reference frequencies [49]. Even if the reference frequencies are similar among individual bats in a group, the Doppler effect and DSC may cause frequency shifts adequate for an individual's auditory fovea to filter vocalizations from other bats. Although the reference frequency can differ every day, bats are unlikely to change their reference frequency to avoid the overlap of the reference frequencies among individuals flying together. The auditory fovea can function as a frequency filter to distinguish bats' own echoes from other bats' sounds.

Counterintuitively, Furusawa *et al*. [33] demonstrated that the reference frequencies of two Japanese horseshoe bats became more similar when they flew together [33]. In the present study, we did not observe any clear pattern of changes in the reference frequencies across flight conditions. One possible reason for this disparity may be the differences in flight tasks. Furusawa *et al*. [33] trained bats to land on a front wall in an experimental room, whereas our bats flew without a particular task [33]. When two bats with similar reference frequencies fly toward the same stable target with DSC to the target, the echo frequencies of one bat and frequency of echoes it receives from another bat could be within the range of its auditory fovea. In such a situation, CF-FM bats could still control the reference frequency to solve jamming problems.

*Miniopterus fuliginosus* increase the individual differences in the terminal frequencies of emitted pulses when flying with other individuals to reduce mutual interferences [9]. Similarly, Hiryu *et al.* demonstrate that *E. fuscus* shift frequencies of the first and the second pulses of paired pulses to overcome pulse-echo ambiguity in cluttered environment [50]. On the other hand, we did not find that *R. f. nippon* changed the reference frequencies in the presence of conspecifics and possibly in the presence of clutter [51]. However, we observed that *R. f. nippon* emphasized the tFM components of the emitted pulses in the presence of the other bats, which is a similar response when FM bats placed in clutter fly in the vicinity of other bats. The present study indicates that the overall problem of flying in any complex scene, whether made complex by obstacles or by other flying bats, is primarily a wideband sonar problem involving localizing the other objects.

In the present study, we recorded pulses and echoes generated by individual bats when three bats were flying together. The bats broadened the bandwidth and lengthened the duration of their tFM component while in group flight. This suggests that they highlight their tFM component to accurately measure distances in the presence of conspecifics. Although the reference frequencies were as precise in the group flight as in the single flight, no clear tendency was observed across the flight conditions. By exhibiting DSC behaviour, the frequency shifts that occur with other bat vocalizations due to the Doppler effect may aid the auditory system, which is sharply tuned to individual-specific reference frequencies to extract weak echoes.

## Data accessibility

The data supporting the findings of this study are available from the Dryad Digital Repository: https://doi.org/10.5061/dryad.v9s4mw6wn [52].
